# Updated transient gene expression protocol in Expi293F cells using PEI

**DOI:** 10.3389/fbioe.2025.1661193

**Published:** 2025-12-03

**Authors:** Gustaf Hederoth, Andrés de la Rosa, Ana Godec, Ximena Aguilar, Antonino Napoleone, Alex Petrovic, Nicole G. Metzendorf, Greta Hultqvist

**Affiliations:** 1 Department of Pharmacy, Uppsala University, Uppsala, Sweden; 2 Department of Public Health and Caring Sciences, Uppsala University, Uppsala, Sweden

**Keywords:** transient gene expression protocol, transfection, protocol update, lab-scale, Expi293F, PEI, salmon sperm DNA, celpure

## Abstract

Transient gene expression (TGE) is commonly used for the rapid production of protein-based therapeutics, including antibodies that require post-translational modifications. We previously published a protocol for efficient and cost-effective TGE of multispecific and multivalent antibodies. Here, we describe an optimized version of this protocol with key improvements in cost, workflow speed, and production capacity. First, the expensive Expi293 expression medium was replaced with BalanCD HEK293 medium, resulting in a substantial decrease in medium-related costs by approximately 90%. The addition of Pluronic F-68 was omitted, as the new medium already contains a similar surfactant. To minimize plasmid DNA usage, salmon sperm DNA was included as filler DNA during transfection, enabling a significant reduction in plasmid DNA input without compromising antibody yield. Second, the harvesting procedure was shortened from 2.5 h to just 15 min by adding the mineral compound diatomaceous earth (Celpure®) to the culture supernatant. This effectively absorbs and sequesters cells and debris, allowing rapid filtration without filter clogging or the previously required 1-h centrifugation step. Finally, we recommend high-flow rate HiScreen Fibro PrismA columns to further accelerate downstream antibody purification. Together, these improvements streamline the TGE workflow in Expi293F cells, enhance scalability, and increase throughput while maintaining efficiency in antibody production.

## Introduction

Antibody-based therapeutics represent a rapidly expanding field, with approximately ten new biopharmaceuticals approved annually by the US Food and Drug Administration and/or the European Medicines Agency since 2001 (Kaplon et al., 2022). Their success is largely due to their high selectivity and strong target binding, which enables the treatment of diseases that small-molecule drugs often fail to address (Stanimirovic et al., 2018). As demand for antibody-based treatments increases, so does the need to improve production efficiency.

While recombinant antibody production in *Escherichia coli* is efficient, prokaryotic expression systems are unsuitable for producing correctly folded, multi-domain eukaryotic antibodies that require endogenous post-translational modifications. To meet these requirements, mammalian cells are commonly used as expression systems (Khan, 2013). For large-scale production of antibody-based drugs, stable cell lines, where the gene of interest is integrated in the genome, are typically used. However, establishing stable cell lines typically requires laborious clonal selection and can take 3 and 12 months (Bandaranayake and Almo, 2014; Cervera et al., 2015; Baldi et al., 2007; Carpentier et al., 2007).

In contrast, transient gene expression (TGE) in mammalian cells is preferred during early-stage drug development, enabling rapid production of candidate antibodies. TGE produces milligram-to gram-scale quantities of antibody per litre of culture within 4–12 days (Jäger et al., 2015; Geisse, 2009).

TGE works by transfecting host cells with one or more vectors containing the gene(s) of interest. These vectors, often plasmids, must reach the host cell nucleus to drive transient expression, as they are not replicated during cell division (Gutiérrez-Granados et al., 2018). While several methods exist for delivering vectors into cells, non-viral approaches are the most commonly used in TGE (Baldi et al., 2007; Jäger et al., 2015). One effective and economical transfection reagent is polyethyleneimine (PEI), which achieves high transfection efficiencies and is significantly less expensive than other reagents such as Lipofectamine™ (Gutiérrez-Granados et al., 2018; Fang et al., 2017).

Among mammalian systems used for TGE, Expi293F cells have become a widely adopted host due to their high-density suspension growth and superior antibody expression capacity. Derived from the human embryonic kidney (HEK293) lineage, Expi293F cells are adapted for serum-free, chemically defined media and can be cultured at volumes suitable for both small-scale and large-scale antibody production. Their compatibility with PEI-based transfection and high volumetric productivity makes them ideal for affordable and scalable production of recombinant antibodies, including complex formats such as bispecific and multivalent constructs (Fang et al., 2017).

**FIGURE 1:**
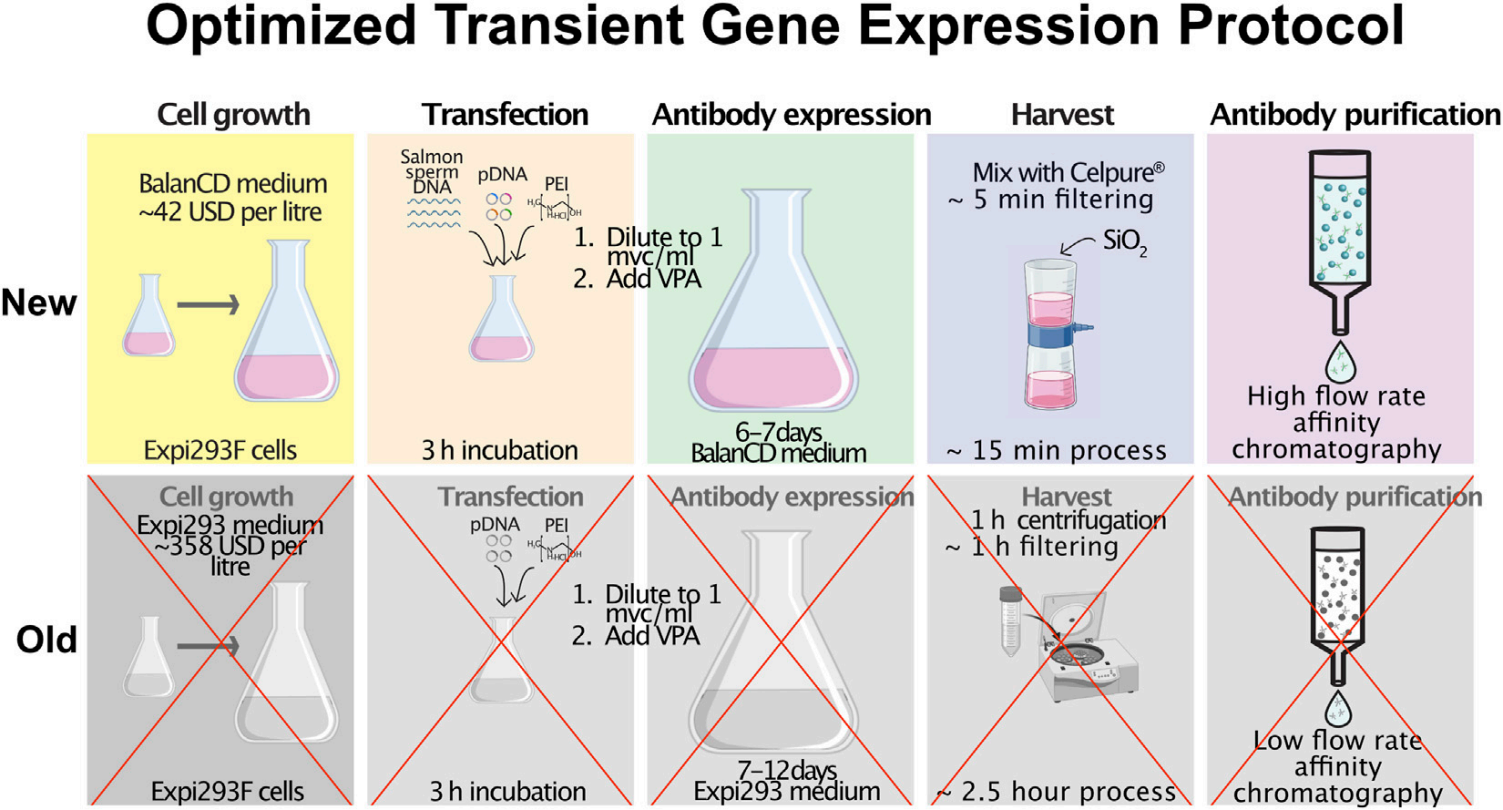
Updated protocol for transient gene expression (TGE) with cost reduction and accelerated harvest process. Cell growth: The costly Expi293 cell medium ($385/L) was replaced with BalanCD HEK293 medium ($42/L), resulting in a substantial decrease in medium-related expenses, a cost reduction of nearly 90%. Pluronic F-68 is no longer added after transfection, as the new medium contains a similar component. Transfection: To reduce the amount of expensive plasmid DNA required, salmon sperm DNA is added as filler DNA, allowing total plasmid usage to be reduced by 80% of the original amount. Harvest: The previous TGE protocol required a 1-h centrifugation step followed by a 1-h filtration step. In the updated protocol, the harvested culture is directly mixed with Celpure®, a diatomaceous earth-based filter aid, enabling rapid filtration and eliminating the need for centrifugation. The total harvest time is reduced from approximately 2.5 h to just 15 min. Purification: Following harvest, antibodies are purified using high-flow rate chromatography, allowing rapid and scalable protein recovery suitable for high-throughput applications.

Although numerous research groups and companies have developed efficient TGE protocols, these are rarely published and often remain inaccessible. To support continued progress in the field, it is essential to share knowledge that enables the optimization of antibody production workflows. This manuscript presents improvements to a previously published and widely cited TGE protocol for the economical production of multispecific and multivalent antibodies (Fang et al., 2017). Our updated method is more resource-efficient, faster, and simpler, while maintaining high antibody yields (Figure 1).

## Materials and methods

### Materials

#### LB medium

LB medium was prepared in deionized water using 0.1% (w/v) NaCl, 0.1% (w/v) tryptone (Sigma/Merck, Darmstadt, Germany, cat. no. 1072131000), and 0.05% (w/v) yeast extract (Sigma/Merck, cat. no. Y1625). The medium was autoclaved and stored at 4 °C until use.

#### Cell media

Cell culture media was prepared by dissolving 2.13% (w/v) of BalanCD HEK293 medium powder (FUJIFILM/Irvine Scientific, Santa Ana, CA, United States, cat. no. 94137) and 11.9 mM of sodium bicarbonate (Sigma/Merck, cat. no. S8875) in deionized water, along with L-glutamine (Thermo Fisher, cat. no. 25030081) at a final concentration of 4 mM. The medium was vacuum-filtered through a 0.22 μm filter (Sigma/Merck, cat. no. GPWP04700) and stored at 4 °C until use.

#### PEI solution

A 1 mg/mL polyethyleneimine (PEI) solution was prepared containing 40 kDa PEI Max powder (Polysciences, Warrington, PA, United States, cat. no. 24765–1) dissolved in deionized water, then vacuum-filtered through a 0.22 μm filter. The PEI solution was stored at −20 °C and thawed at room temperature prior to use.

#### Salmon sperm DNA

UltraPure Salmon sperm DNA solution (Invitrogen, cat. No. 15632011) was used at a stock concentration of 10 mg/mL.

#### VPA solution

A 0.5 M solution of VPA (2-propyl-pentanoic acid, sodium salt; Sigma/Merck cat. no P4543) was prepared in deionized water, vacuum-filtered through a 0.22 μm filter, and stored at −20 °C. The solution was thawed at room temperature prior to use.

## Methods

### Cloning

The heavy and light chains genes were cloned into separate pcDNA3.4 TOPO expression vectors by GeneArt (ThermoFisher GeneArt, Regensburg, Germany). Gene synthesis and cloning were performed by GeneArt using ThermoFisher’s recommended Kozak consensus sequence and codon optimization for human cells, as determined by the GeneArt codon optimization tool. The commercially available pcDNA3.4 vector is designed for high-level expression in mammalian systems and includes the human cytomegalovirus (CMV) promoter to drive strong transcriptional activity. Additionally, the vector contains the woodchuck hepatitis virus posttranscriptional regulatory element (WPRE) to enhance transcript stability and expression efficiency.

Both heavy and light chain constructs included their native signal peptides to ensure proper secretion of the expressed antibody into the culture medium.

### Amplification and purification of plasmid DNA

Plasmid DNA was amplified in *Escherichia coli* One Shot Top™ TOP10 chemically competent cells (ThermoFisher Scientific, Waltham, MA, cat. no. K204040) according to the manufacturer’s protocol. Following bacterial culture and plasmid amplification, plasmid DNA was purified using GeneJET endo-free plasmid maxiprep kit (ThermoFisher, cat. no. K0861) following the kit instructions.

### Cell thawing and culturing

Expi293F cells (Thermo Fisher Scientific, cat. no. A14527) were thawed and cultured in BalanCD HEK293 medium (Fujifilm Irvine Scientific, cat. no. 94155), a more affordable alternative to the Expi293 Expression Medium recommended by the manufacturer (Expi293 Expression System, 2025). A direct medium exchange was performed without compromising cell viability, as previously reported (Napoleone et al., 2021). According to the supplier’s protocol, Expi293F cells are typically cultured in an 8% CO_2_ atmosphere to maintain pH in the proprietary medium. However, in this protocol, cells were adapted to standard 5% CO_2_ conditions without affecting viability or productivity, as BalanCD HEK293 medium is compatible with lower CO_2_ levels.

Cryopreserved cells were stored at −150 °C and thawed rapidly in a 37 °C water bath until dissolved. The vial was decontaminated with 70% ethanol before opening in a laminar flow hood. Cells were gently transferred to 5 mL of pre-warmed BalanCD HEK293 medium, centrifuged at 200 *g* for 5 min to remove residual DMSO, and resuspended in 1 mL of fresh pre-warmed medium. The suspension was transferred to a 125 mL sterile, vented Erlenmeyer flask containing 30 mL of BalanCD HEK293 medium and incubated at 37 °C, ≥80% humidity, and 5% CO_2_ on an orbital shaker. Shaking speed was adjusted based on the platform diameter: 125 rpm (19 mm), 120 rpm (25 mm), or 95 rpm (50 mm). Cell viability was monitored post-thaw and typically reached ≥90% within 3–4 days. Cells were subcultured when they reached 1 – 3 million viable cells/mL (mvc/mL).

For routine maintenance, cells were cultured at 37 °C with 5% CO_2_ in 30 mL volumes in 125 mL Erlenmeyer plastic flasks with ventilated caps (VWR, cat. no. 734–1832). Cultures were maintained between 0.3 and 3 – 5 mvc/mL and passaged every 3–4 days. Cell viability and viable cell density were assessed using trypan blue exclusion and measured with a Countess 3 automated cell counter (Invitrogen).

### Cryopreservation of Expi293F™ cells

Expi293F cells were cryopreserved at a density of 1 × 10^7^ viable cells/mL in BalanCD HEK293 medium supplemented with 10% DMSO. Cells were pelleted, resuspended in ice-cold freeze medium, and frozen using a controlled-rate freezing method (−1 °C/min), followed by transfer to −150 °C for long-term storage.

### Day before transfection

To maximize transfection efficiency, Expi293F cells were split the day before transfection to remove depleted medium and accumulated secreted factors, and to ensure the culture was in a healthy logarithmic growth phase. Cells were harvested by centrifugation at 200 *g* for 10 min at room temperature. The spent medium was carefully removed, and cells were resuspended in pre-warmed fresh BalanCD HEK293 medium at a density of 1 mvc/mL.

Alternatively, cells can be resuspended at 2.5 – 3 mvc/mL to reduce medium usage without compromising transfection efficiency or protein yield. Following resuspension, cells were returned to standard culture conditions (37 °C, 5% CO_2_, 125 rpm) and incubated overnight prior to transfection.

### TGE transfection and harvesting

On the day of transfection, Expi293F cell density and viability were measured. Cells were harvested by centrifugation at 200 *g* for 10 min at room temperature and resuspended in pre-warmed BalanCD HEK293 medium to a final density of 20 mvc/mL.

Transfections were performed in 20 mL cultures (at 20 mvc/mL) by adding a total of 1.25 μg DNA per mvc, using a 1:5 ratio of plasmid DNA to salmon sperm DNA. Polyethyleneimine (PEI MAX, linear 40 kDa, 1 mg/mL) was added at 3.75 µL per mvc, corresponding to an optimized 3:1 PEI-to-DNA ratio (de los Milagros Bassani Molinas et al., 2014). Transfections were carried out in 125 mL Erlenmeyer plastic flasks with ventilated caps (VWR, cat. no. 734–1832) and incubated for 3 h under standard culture conditions (37 °C, 5% CO_2_, 125 rpm).

After 3 h, transfected cells were diluted to final density of 1 mvc/mL in 400 mL of BalanCD HEK293 cell medium supplemented with valproic acid (VPA) to a final concentration of 3.5 mM. Cultures were transferred to 1 L Erlenmeyer plastic flasks with ventilated caps (VWR, cat. no. 734–1889) and incubated for 6–7 days under standard shaking conditions.

For harvesting, Celpure® P300 (Sigma/Merck, cat. no. 525243) was added directly to the cell culture at 1% (w/v). The suspension was briefly mixed and then vacuum-filtered through a 0.22 μm filter to remove cells and debris prior to purification.

### Antibody purification

The purification strategy was dependent on the specific design of the protein construct and the presence or absence of affinity tags, therefore, protocols were tailored accordingly and are not described in full detail here.

However, for constructs containing Fc regions, we recommend using HiScreen Fibro PrismA columns (Cytiva, cat. no. 17549816) as an efficient alternative to traditional HiTrap Protein-G columns (Cytiva, cat. no. 17040501). HiScreen Fibro PrismA columns support flow rates up to 30 mL/min, compared to 5 mL/min for Protein-G columns, enabling up to an 80% reduction in purification time without compromising yield or purity. Elution was performed using 0.7% (v/v) acetic acid, a milder elution condition that minimizes antibody denaturation while maintaining efficient recovery. Eluted fractions were immediately neutralized by the addition of with 1 M Tris, pH 8.0, at a ratio of 1:10 (v/v) to ensure pH stability and maintain antibody integrity.

### Western blot

Equal volumes of representative bispecific antibody samples, collected directly from cell culture supernatant or from purified protein preparations, depending on specific experiment, were mixed with 4x LDS sample buffer (ThermoFisher, cat. no. B0007) and loaded onto 4%–12% Bis-Tris protein gels (Invitrogen, cat. no. NW04125BOX) covered in MES buffer (ThermoFisher, cat. no. NP0002). The samples were separated on SDS-PAGE with 80 V for approximately 2 h, then transferred to a methanol activated PVDF membrane (ThermoFisher, cat. no. 88520) at 100 V for 2 h. After transfer, membranes were dried, reactivated in methanol, and blocked in 5% (w/v) skimmed milk in TBS-T overnight. The membrane was incubated with a goat-anti-mouse conjugated to horse-radish peroxidase (HRP) (Sigma, cat. no. 12–349). The membrane was washed thrice with TBS-T. HRP chemiluminescent substrate (Invitrogen, cat. no. WP20005) was added and imaged using the Odyssey Fc (LI-COR Biosciences).

## Results

### Updated TGE protocol with resource-efficient media, reduced plasmid DNA input, and accelerated harvesting procedure

Expi293F cells used in this study are HEK293 cells adapted to grow in suspension at high density (>5 mvc/mL), enabling significantly higher recombinant protein yields per culture volume (Expi293 Expression System, 2025). In our previously published TGE protocol (Fang et al., 2017), we used the recommended Expi293F expression medium, which is specifically formulated for these cells but is relatively expensive. To reduce costs, we replaced it with BalanCD HEK293 medium, which decreased the medium cost from $358 to $42 per litre, a reduction by nearly eightfold. BalanCD HEK293 medium was selected as a replacement because it is specifically designed to support high-density, suspension-cultured HEK293 cells and enhance TGE performance (Gao et al., 2022).

Expi293F cells adapted to BalanCD HEK293 medium without requiring a stepwise adaptation process and performed comparably to cells cultured in Expi293 expression medium with respect to viability, cell density, growth rate, and recombinant protein yield, as previously shown (Napoleone et al., 2021). Based on these results, BalanCD HEK293 medium was adopted as the expression medium in the updated protocol. Furthermore, because BalanCD HEK293 medium already contains 0.1% poloxamer (a surfactant similar to Pluronic F-68), the previously required Pluronic F-68 supplementation was no longer necessary (Vasu et al., 2025).

**FIGURE 2:**
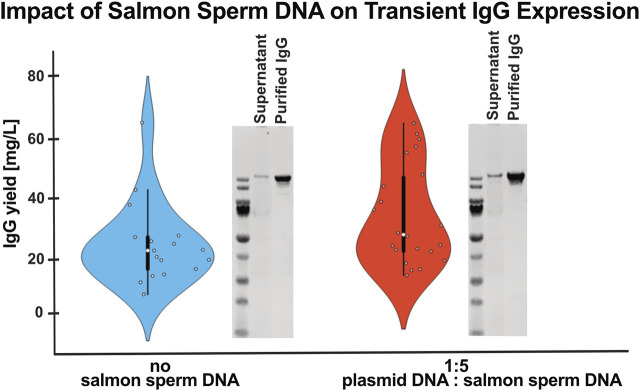
Reduced plasmid input using salmon sperm DNA does not compromise IgG yield or integrity. Total IgG yields after purification are shown for different antibody constructs transfected into Expi293F cells with either 100% plasmid DNA (1.25 µg/mvc) or with a 1:5 ratio of plasmid DNA to salmon sperm DNA, while keeping total DNA amounts and expression volumes constant. Data is presented as a violin plot, showing the distribution and variability across constructs. Comparable yields [mg/mL] between conditions indicate that plasmid DNA input can be substantially reduced without compromising production efficiency. Number of independent transfections: no salmon sperm DNA (n = 17); with salmon sperm DNA (n = 21). To the right of the violin plots, a representative Western blot shows an bispecific antibody in the cell culture supernatant and after purification, under non-reducing conditions. The integrity of the full-length bispecific antibody is maintained in both transfection conditions, confirming that the use of filler DNA does not negatively impact antibody expression or quality.

Plasmid DNA production is both time-consuming and expensive, making its reduction a valuable improvement. By maintaining the total DNA concentration while diluting the plasmid DNA with inexpensive salmon sperm filler DNA, plasmid usage was reduced by up to 80%. As shown in Figure 2, two transfection conditions using the same total DNA concentration, one with and one without salmon sperm DNA, produced comparable antibody yields, demonstrating that filler DNA does not negatively impact expression efficacy. Additionally, we included a representative Western blot of purified IgG, confirming that there was no difference in antibody quality or integrity after purification between the conditions (Figure 2).

### Streamlined harvesting using Celpure® eliminates centrifugation and prevents filter clogging

In our previous TGE protocol, the harvesting procedure required a 1-h centrifugation step to pellet cells and debris, followed by a 1-h filtration process. Despite centrifugation, 0.22 µm filters frequently clogged and had to be replaced 3–5 times due to residual cell material. To overcome this bottleneck, Celpure® P300, a refined diatomaceous earth, was incorporated into the updated TGE protocol. Celpure® is a natural siliceous mineral compound with high absorptive capacity for water and oils (Özen et al., 2015), enabling it to effectively sequester cells and cell debris. This allows for direct filtration of the culture supernatant without prior centrifugation (Buyel et al., 2015).

After mixing the harvested medium with Celpure®, filtration proceeds rapidly and without clogging, eliminating the need to replace filters. This modification reduced filtration time sixfold, from 60 min to just 10 min, and shortened the total harvest time from approximately 2.5 h to just 15 min.

**FIGURE 3:**
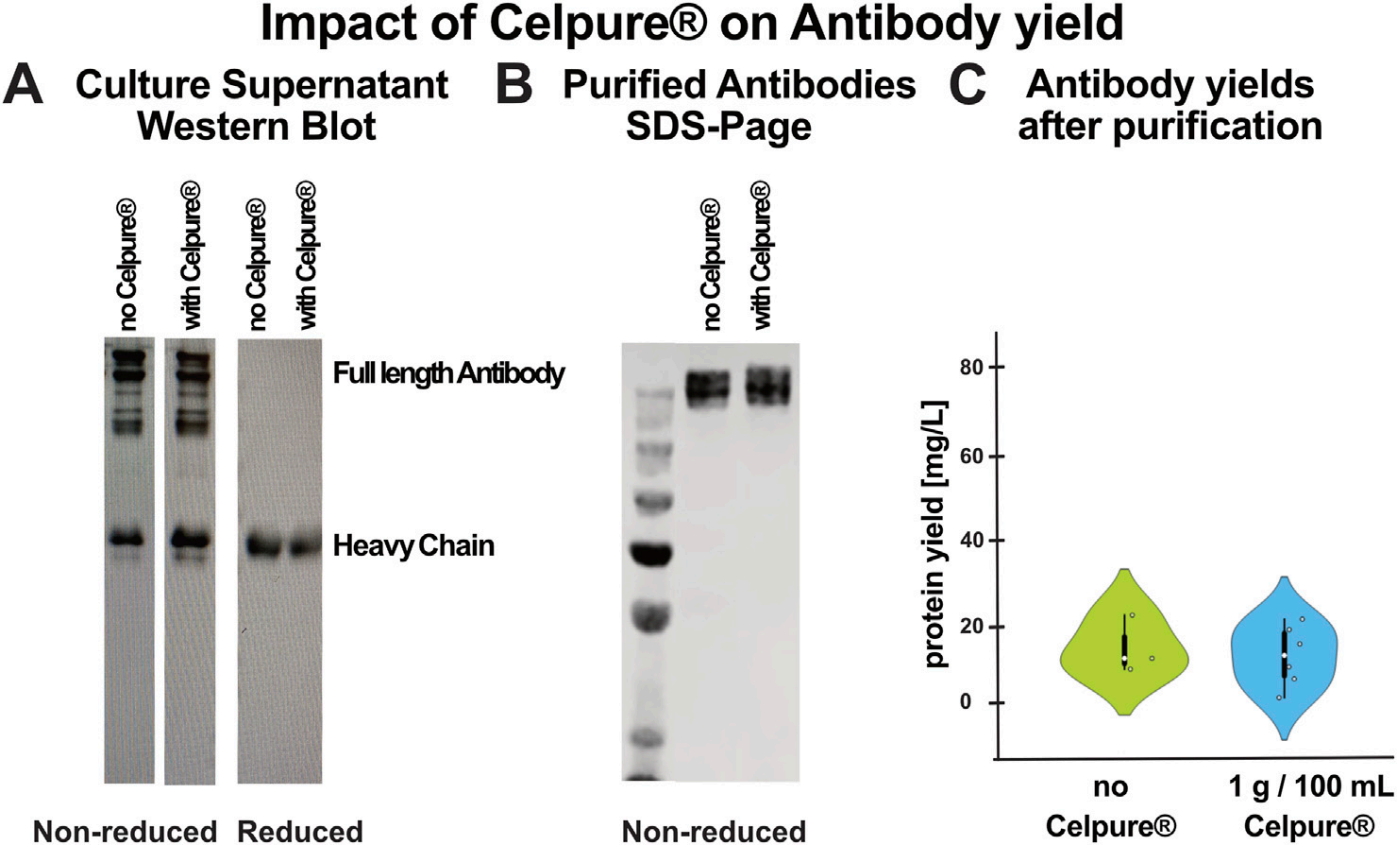
Celpure® treatment does not affect antibody recovery or integrity. **(A)** Western blot analysis of a representative bispecific antibody in culture supernatants with and without Celpure® treatment, analysed under reducing and non-reducing conditions. Under non-reducing conditions, multiple bands reflect different disulfide-bonded conformations, such as intact antibodies, dimers, or partially assembled species. Upon DTT reduction, these resolved into individual heavy and light chains; the detection antibody, being specific for full-length antibodies or heavy chains of native mouse IgG, detected only the heavy chain, resulting in a single band. Comparable band intensities between treated and untreated samples indicate that Celpure® does not interfere with antibody expression or recovery. **(B)** SDS-PAGE analysis of purified antibodies from TGE supernatants, with or without Celpure® treatment. Same samples were run under non-reducing conditions. The banding patterns are indistinguishable between conditions, indicating that Celpure® does not affect antibody integrity during purification. **(C)** Total antibody yields after purification using HiTrap Protein-G columns from TGE supernatants, either untreated (green, n = 4) or treated with 1 g Celpure® per 100 mL supernatant (blue, n = 6). Data is presented as violin plots, showing the distribution and variability across the constructs. Protein yields remained consistent across conditions, confirming that Celpure® does not negatively impact purification efficiency.

Importantly, the updated harvesting procedure had no negative impact on antibody yield. As shown in Figure 3A, Western blot analysis confirmed that Celpure® treatment does not interfere with antibody expression or recovery. Under non-reducing conditions, multiple bands were observed, corresponding to different disulfide-bonded conformations, such as intact antibodies, dimers, or partially assembled species. Upon DTT reduction, these resolved into individual heavy and light chains. The detection antibody, specific for full-length antibodies or heavy chains of native mouse IgG, recognised only the heavy chain, resulting in a single band (Figure 3A). To further validate purification, an SDS-PAGE image of the purified IgG was included (Figure 3B). Additionally, total antibody levels remained consistent between Celpure®-treated and untreated samples, confirming that the treatment does not affect antibody yield (Figure 3C).

**FIGURE 4:**
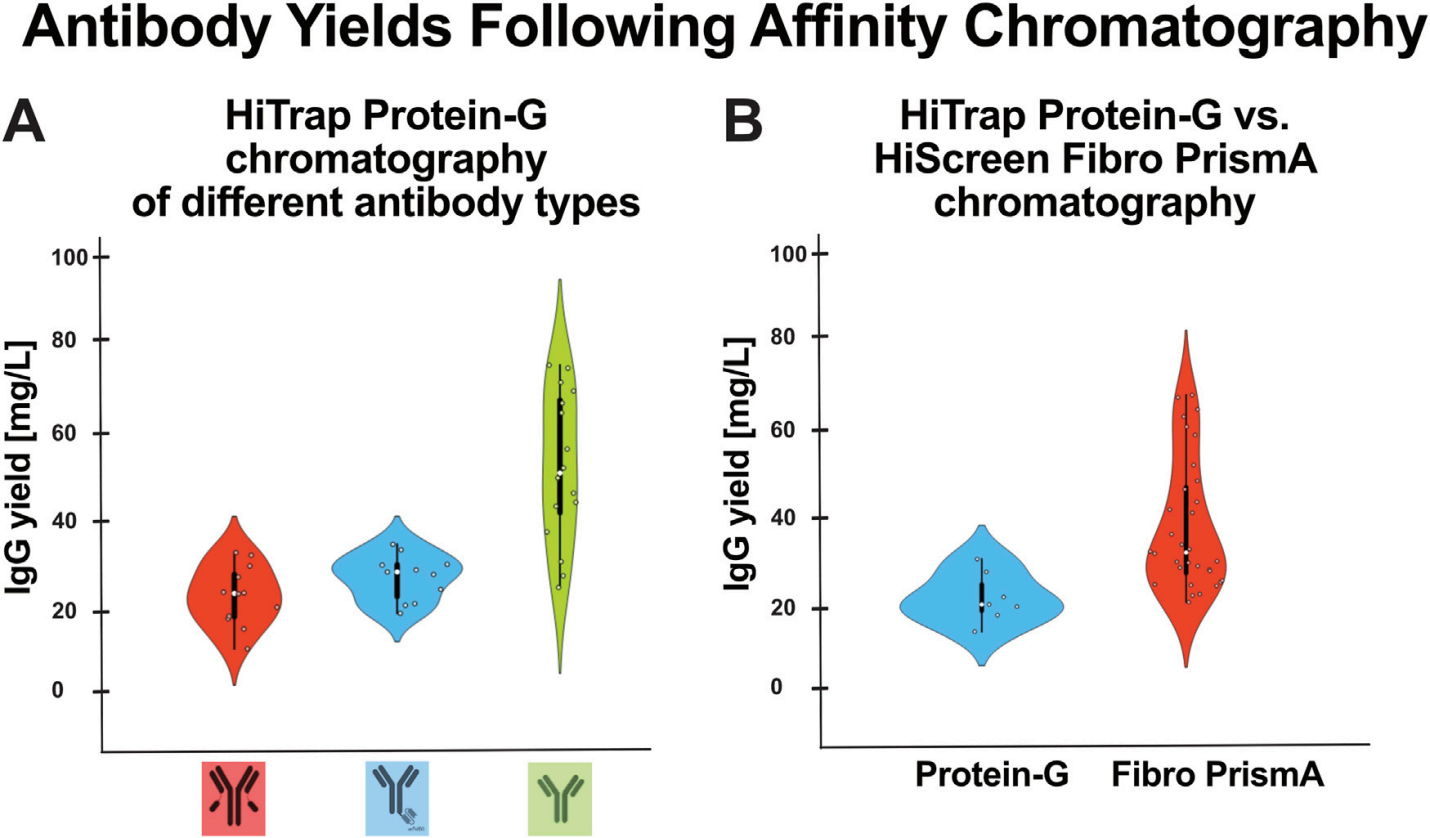
Comparison of IgG yields across antibody formats and purification methods. **(A)** Antibody yields of various antibody formats purified using HiTrap Protein-G chromatography. Bispecific antibodies (red, n = 11 and blue, n = 11) consistently showed lower protein yield compared to traditional IgG constructs (green, n = 16). **(B)** Comparison of purification methods for representative bispecific antibodies using HiTrap Protein-G (blue, n = 7) with HiScreen Fibro PrismA columns (red, n = 27). The HiScreen Fibro PrismA column, which supports higher flow rates, tended to achieve higher protein recovery for bispecific formats.

To evaluate the impact of antibody format on production efficiency, we compared antibody yields across different antibody types purified using HiTrap Protein-G chromatography. As shown in Figure 4A, bispecific antibodies consistently yielded lower amounts of purified antibody compared to conventional IgG constructs, suggesting that antibody design may influence expression levels or purification efficiency.

To address this limitation, we evaluated an alternative purification method using the high-flow HiScreen Fibro PrismA column. As shown in Figure 4B, the bispecific antibody yielded even higher amounts of purified antibody when processed using the HiScreen Fibro PrismA column compared to traditional HiTrap Protein-G. This suggests that the sixfold increased flow rate and improved binding capacity of HiScreen Fibro PrismA can enhance purification efficiency, particularly for more complex antibody formats.

Collectively, these modifications to both upstream and downstream workflows have made the TGE protocol (Figure 1) significantly more resource-efficient, streamlined, and faster, while maintaining high antibody yield, integrity, and transfection efficiency.

## Discussion

We report an improved transient gene expression (TGE) protocol that enhances the efficiency and affordability of producing multispecific and multivalent antibodies, extending our earlier work (Fang et al., 2017). The new protocol is faster and significantly more cost-efficient than the original, while still achieving effective protein yields with optimal PEI and DNA ratios (de los Milagros Bassani Molinas et al., 2014). Our earlier TGE method has been widely cited, likely due to its ability to reduce production costs by replacing the expensive transfection reagent ExpiFectamine™ 293 with inexpensive and broadly available reagents (ExpiFectamineTM 293, 2025).

This updated protocol further lowers costs by substituting the high-cost Expi293 expression medium with the substantially cheaper BalanCD HEK293 medium, resulting in a significant reduction in medium-related costs. While similar optimizations have been implemented by other groups and commercial providers, they are rarely published, limiting their accessibility to the broader research community. Our work aims to fill that gap by providing an openly available tested alternative. The updated TGE protocol is not intended to reach gram-per-litre titers achievable with engineered CHO or HEK293 cell lines utilizing enhancers, temperature shifts, and extended fed-batch processes (Rajendra et al., 2015). Our Expi293F-based transient transfection protocol provides a simple, flexible, and economical platform for antibody production, comparable in scope to optimized CHO-based systems previously described (Schmitt et al., 2020). While CHO cells remain the gold standard for industrial-scale production and regulatory compliance, HEK293 cells offer advantages for rapid screening, ease of transfection, and consistent yields without the need for extensive cell line development. Each system has unique strengths, and the choice depends on the intended application and production scale.

Simplification of the process was achieved by removing Pluronic F-68, since BalanCD HEK293 medium already contains 0.1% poloxamer, which provides the same protective function. In addition, most of the plasmid DNA was replaced with salmon sperm DNA, a low-cost, inert filler (Rajendra et al., 2012; Carreño et al., 2024; Kichler et al., 2005); alternative carrier DNAs, such as herring DNA, can also be used (Carreño et al., 2024; Pradhan and Gadgil, 2012). Importantly, the addition of salmon sperm DNA did not negatively affect protein titer despite an 80% reduction in plasmid DNA (Figure 2). This observation is consistent with previous studies, which have similarly reported no significant changes in protein expression levels upon substitution with inert carrier DNA (Rajendra et al., 2012; Kichler et al., 2005).

To further enhance the practicality of the protocol, we streamlined the harvesting step by incorporating Celpure®, a diatomaceous earth-based filter aid (Buyel et al., 2015). Celpure® effectively sequesters cells and cell debris, enabling rapid filtration without centrifugation or frequent filter replacement. This modification reduced the harvest time from approximately 2.5 h to just 15 min, without negatively affecting yield or product integrity, as confirmed by Western blot analysis (Figure 3A) and total protein recovery data (Figure 3B) and thus representing a substantial improvement in workflow efficiency.

PEI transfection performance is highly sensitive to numerous parameters, including PEI molecular weight and structure, DNA quality, PEI:DNA ratio, cell density at transfection, media composition, and the specific HEK293 variant used (Baldi et al., 2007; Rajendra et al., 2012; Geisse et al., 2005; Backliwal et al., 2008a; Tom et al., 2008; Bollin et al., 2011; Budge, 2025; Elgundi et al., 2017). Studies reporting high PEI-mediated titers typically rely on extensively optimized conditions such as high-density transfection or serum-free suspension cultures, tailored feeding strategies, or the inclusion of expression enhancers, all of which can significantly increase productivity (Budge, 2025; Elgundi et al., 2017; Backliwal et al., 2008b; Tuvesson et al., 2008). In contrast, standard PEI protocols, or those using different culture formats and media conditions often yield lower titers, consistent with the levels observed in our study. In our work, we used an optimized 3:1 PEI-to-DNA ratio (de los Milagros Bassani Molinas et al., 2014) and obtained antibody yields of 20–80 mg/L (Figure 4) that are comparable to several other reports employing similar PEI-based transient expression systems, 10 mg/L (Backliwal et al., 2008b; Baldi et al., 2005) (Blackliwal et al., 2007) and 25 mg/L (Baldi et al., 2007).

We also examined the impact of antibody format on yield and purification efficiency. As shown in Figure 4A, bispecific antibodies exhibited lower yields compared to conventional IgG constructs when purified using Protein-G chromatography. However, the use of HiScreen Fibro PrismA columns, which support higher flow rates, improved recovery for bispecific constructs (Figure 4B), suggesting that chromatography selection can mitigate format-specific yield limitations. In addition to the HiTrap Protein-G and HiScreen Fibro PrismA resins evaluated in this study, a range of other high-performance affinity media such as Protein A, Protein L, and CaptureSelect resins are available and may offer distinct advantages depending on the antibody format or application. While a comprehensive comparison of these resins was beyond the scope of our current work, future studies could explore their performance, especially for non-IgG constructs or challenging purification scenarios.

This improved and accessible TGE protocol is particularly well suited for the production of complex antibody formats, including bispecific and multivalent constructs, which often present additional challenges in yield and stability. By minimizing reliance on costly proprietary reagents and accelerating key steps such as transfection and harvest, the protocol enables rapid and scalable antibody production, ideal for high-throughput screening and early-stage therapeutic development. Future optimization efforts may focus on adapting the workflow to other expression systems or further enhancing yields for structurally demanding antibody formats.

## Data Availability

The raw data supporting the conclusions of this article will be made available by the authors, without undue reservation.
